# Engagement of the CXCL12–CXCR4 Axis in the Interaction of Endothelial Progenitor Cell and Smooth Muscle Cell to Promote Phenotype Control and Guard Vascular Homeostasis

**DOI:** 10.3390/ijms23020867

**Published:** 2022-01-14

**Authors:** Sebastian F. Mause, Elisabeth Ritzel, Annika Deck, Felix Vogt, Elisa A. Liehn

**Affiliations:** 1 Department of Internal Medicine I, Cardiology, University Hospital Aachen, RWTH Aachen University, 52074 Aachen, Germany; annika.deck@md.nord.de (A.D.); fvogt@ukaachen.de (F.V.); eliehn@ukaachen.de (E.A.L.); 2 Institute for Molecular Cardiovascular Research (IMCAR), University Hospital Aachen, RWTH Aachen University, 52074 Aachen, Germany; Elisabeth.Ritzel@rwth-aachen.de; 3 Department of Otorhinolaryngology Head and Neck Surgery, Klinikum Stuttgart, 70174 Stuttgart, Germany; 4 Institute of Molecular Medicine, University of South Denmark, DK-5000 Odense, Denmark; 5 Victor Babes National Institute of Pathology, 050096 Bucharest, Romania

**Keywords:** endothelial progenitor cells, smooth muscle cells, CXCR4, chemokine, atherosclerosis, vascular injury, neointima, restenosis

## Abstract

Endothelial progenitor cells (EPCs) are involved in vascular repair and modulate properties of smooth muscle cells (SMCs) relevant for their contribution to neointima formation following injury. Considering the relevant role of the CXCL12–CXCR4 axis in vascular homeostasis and the potential of EPCs and SMCs to release CXCL12 and express CXCR4, we analyzed the engagement of the CXCL12–CXCR4 axis in various modes of EPC–SMC interaction relevant for injury- and lipid-induced atherosclerosis. We now demonstrate that the expression and release of CXCL12 is synergistically increased in a CXCR4-dependent mechanism following EPC–SMC interaction during co-cultivation or in response to recombinant CXCL12, thus establishing an amplifying feedback loop Additionally, mechanical injury of SMCs induces increased release of CXCL12, resulting in enhanced CXCR4-dependent recruitment of EPCs to SMCs. The CXCL12–CXCR4 axis is crucially engaged in the EPC-triggered augmentation of SMC migration and the attenuation of SMC apoptosis but not in the EPC-mediated increase in SMC proliferation. Compared to EPCs alone, the alliance of EPC–SMC is superior in promoting the CXCR4-dependent proliferation and migration of endothelial cells. When direct cell–cell contact is established, EPCs protect the contractile phenotype of SMCs via CXCL12–CXCR4 and reverse cholesterol-induced transdifferentiation toward a synthetic, macrophage-like phenotype. In conclusion we show that the interaction of EPCs and SMCs unleashes a CXCL12–CXCR4-based autoregulatory feedback loop promoting regenerative processes and mediating SMC phenotype control to potentially guard vascular homeostasis.

## 1. Introduction

The multifaceted process of atherosclerotic intimal expansion and remodeling after vascular injury with subsequent progressive restenosis and target lesion failure constitutes a major limitation of therapeutic revascularization and are a relevant cause of morbidity and mortality worldwide [1,2]. Given their distinctive plasticity allowing phenotypic transformation with stimulated synthesis of extracellular matrix and their capacity to proliferate and migrate, vascular smooth muscles cells (SMCs) are attributed a dominant but also ambivalent role in atherosclerotic lesion development and neointima formation [3,4,5,6]. Environmental cues and interaction with other residential and recruited cells critically regulate phenotypic transitions and functional properties of SMCs and finally determine a beneficial or detrimental impact on postinjury recovery, as well as the progression and stability of atherosclerotic lesions [7].

CXCR4 and its canonical ligand CXCL12 are substantially engaged in processes following vascular stress and contribute to the establishment of a microenvironment permissive and supportive for regenerative processes including the proliferation and migration of residential vascular cells as well as the recruitment of progenitor cells [8]. The capacity of the CXCL12–CXCR4 axis to shift the inflammatory state of the vessel wall and the phenotype of its constituting cells together with the competence to assist in the maintenance of endothelial integrity further demonstrates its relevance for vascular homeostasis [9,10]. Of note, the role of the CXCL12–CXCR4 axis in native and accelerated, injury-driven atherosclerosis is ambivalent and highly context- and cell-dependent and may therefore confer protective but also aggravating effects on lesion formation [8,11]. Exemplarily, ApoE^−/−^mice repopulated with CXCR4-deficient bone marrow displayed reduced lesions in injury-driven atherosclerosis but exacerbated lesion formation in lipid-driven atherosclerosis [12,13]. Remarkably, the SMC lesion density was reduced in both scenarios. Similarly, SMC accumulation in murine lesions was reduced in a hyperlipidemia-model with SMC-specific CXCR4 deletion and in an injury-model with endothelial-specific CXCR4 deletion [9,14].

As a key regulator for the mobilization of stem and progenitor cells into peripheral blood and their subsequent recruitment to sites of tissue damage or stress, the CXCL12–CXCR4 axis is also crucially involved in the trafficking of endothelial progenitor cells (EPCs) [10,15]. In response to vascular injury, mobilized EPCs recruit to sites of endothelial denudation, accelerate endothelial recovery, and attenuate neointimal formation [16,17]. Inhibition of CXCR4 was shown to impair the regenerative potential of infused EPCs and overexpression of CXCR4 on EPCs following gene transfer was associated with enhanced CXCL12-mediated migration and adhesion of EPCs in vitro and improved endothelial recovery after carotid injury in vivo [18,19].

We have recently shown that EPCs may modulate the phenotype of SMCs, stimulate their proliferation and migration and finally increase their neointimal accumulation following vascular injury [20]. Considering that both cell types not only release CXCL12 and express CXCR4 but also implement sender and receiver functions for the CXCL12–CXCR4 signaling cascade with its double-edged role in injury-induced restenosis and atherosclerosis, we now sought to analyze the role of the CXCL12–CXCR4 axis in various modes of EPC–SMC interaction including migration, proliferation and phenotype control.

## 2. Results

### 2.1. Expression and Release of CXCL12 by SMCs and EPCs

**Figure 1:**
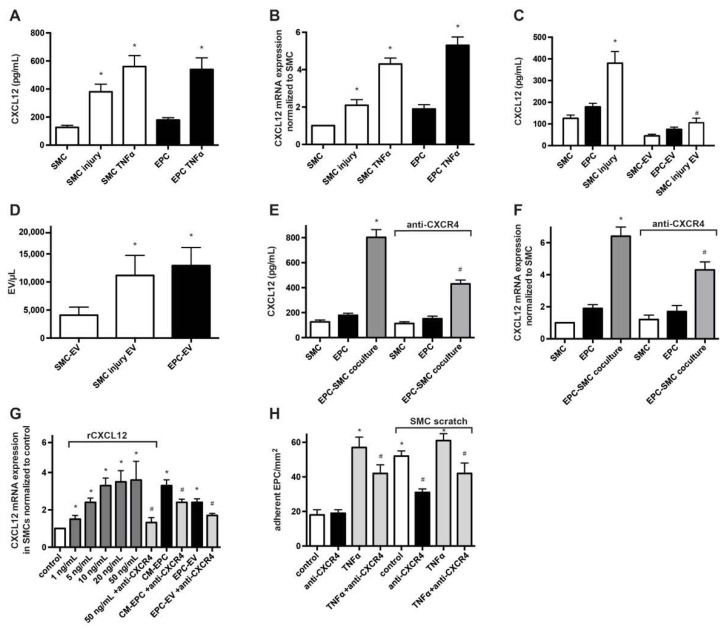
Expression and release of CXCL12 by SMCs and EPCs. (**A**) Analysis of CXLC12 release using ELISA. Cell supernatants from monocultured SMCs and EPCs, treated as indicated, were collected 24 h after cultivation. * *p* < 0.05 vs. untreated cells; *n* = 6. (**B**) Real-time RT-PCR analysis of CXCL12 expression SMCs and EPCs treated as indicated. Results were normalized to CXCL12 expression in SMCs. * *p* < 0.05 vs. untreated cells; *n* = 5. (**C**) Detection of CXCL12 in MVs derived from monocultured SMCs and EPCs. Isolated MVs were lysed in RIPA buffer and CXCL12 levels were determined using ELISA. * *p* < 0.05 vs. non-injured SMCs, # *p* < 0.05 vs. SMC-MV; *n* = 5. (**D**) Enumeration of MVs in the supernatant of EPCs, non-injured SMCs and injured SMCs using flow cytometry with calibrated microbeads. * *p* < 0.05 vs. non-injured SMCs; *n* = 4. (**E**) Evaluation of the effect of EPC-SMC co-cultivation and engagement of CXCR4 on the release of CXCL12. Supernatants from monocultured SMCs, monocultured EPCs and EPCs co-cultured with SMCs, each in the presence or absence of a blocking CXCR4 Ab, were analyzed for CXCL12 concentration using ELISA. * *p* < 0.05 vs. SMCs, # *p* < 0.05 vs. EPC-SMC co-culture in the absence of anti-CXCR4; *n* = 5. (**F**) Real-time RT-PCR analysis of CXCL12 expression to test the impact of EPC-SMC co-cultivation and involvement of CXCR4. CXCL12 transcripts were determined in SMCs, EPCs and EPC-SMC co-cultures in the presence or absence of a blocking CXCR4 Ab. * *p* < 0.05 vs. SMCs, # *p* < 0.05 vs. EPC-SMC co-culture in the absence of anti-CXCR4; *n* = 5. (**G**) Real-time RT-PCR analysis of CXCL12 expression in SMCs treated with various doses of rCXCL12, CM-EPC or EPC-MV in the presence or absence of an anti-CXCR4 Ab. * *p* < 0.05 vs. untreated SMCs (control), # *p* < 0.05 vs. respective treatment in the absence of anti-CXCR4; *n* = 5. (**H**) Adhesion of EPCs to SMCs under flow conditions in vitro. EPCs pretreated with/without an anti-CXCR4 Ab were perfused in a parallel flow chamber and the number of cells EPCs adherent to the SMC monolayer was determined and expressed as adherent cells per 1 mm². For some experiments, the SMC monolayer was wounded by a linear scratch before perfusion of EPCs. * *p* < 0.05 vs. untreated and non-scratched SMCs (control), # *p* < 0.05 vs. respective treatment in the absence of anti-CXCR4; *n* = 4 to 6.

Given the relevance of CXCL12 in the recruitment of progenitor cells, vascular repair and postinjury remodeling as well as primary and secondary atherosclerosis [8], we investigated the pattern of CXCL12 expression and release by EPCs and SMCs not only in monoculture but also following co-cultivation allowing EPC–SMC interaction. Using ELISA, we confirmed that cultured SMCs and particularly EPCs constitutively express and release substantial amounts of CXCL12 (Figure 1A,B). To evaluate the effect of SMC injury on CXLC12 release, we applied multiple linear scratches on the SMC monolayer to cause disruption in the SMC network. Compared to non-injured SMCs, we detected intensified transcription of CXCL12 in SMCs as well as significantly higher CXCL12 concentrations in the supernatant of mechanically injured SMCs, suggesting a stress-triggered induction of CXCL12 (Figure 1A,B). Of note, inflammatory activation of cells with recombinant low-dose TNFα strongly amplified expression and secretion of CXCL12 in SMCs and EPCs. As we have previously observed that EPC-derived microvesicles (MVs, also known as microparticles or extracellular vesicles) alone may modulate key functions of SMCs and as MVs harbor and transfer bioactive substances including chemokines and microRNAs [20,21,22], we also evaluated the presence of CXCL12 in MVs. Indeed, a notable amount of CXCL12 could be identified in MVs derived from EPCs (EPC-MV) and SMCs (SMC-MV), indicating that a relevant portion of released CXLC12 is transferred to MVs (Figure 1C). Enumeration of MVs and cell-number adjusted analysis revealed that formation of MVs is roughly three times more frequent in EPCs than in quiescent SMCs, whereas the relative share of CXCL12 transfer into secreted MVs is rather similar with 42% for EPCs and 36% for SMCs (Figure 1D). The induction of SMC injury led to an almost threefold increase in MV generation with a corresponding enhancement of total CXCL12 content present in SMC-MV (Figure 1C,D).

In a recent study, we have shown that heterocellular interaction of EPCs and SMCs results in the alteration of the secretion profile with a selective positive synergy of co-cultivation for certain factors [20]. Following co-incubation of EPCs and SMCs, we measured not only significantly higher CXCL12 protein levels compared to the respective supernatants of EPC and SMC monocultures but also over-additive absolute values of CXCL12 (803 ± 61 pg/μL) when related to the combined supernatant derived from both monocultures (305 ± 31 pg/μL), indicating a positive synergy of co-cultivation for the release of CXCL12 (Figure 1E). Interestingly, inhibition of the CXCL12 receptor CXCR4 using a blocking Ab or the CXCR4 receptor antagonist AMD3100 (not shown) relevantly reduced the increase in CXCL12 release in co-cultivation experiments. Corresponding results were found when analyzing CXCL12 transcripts (Figure 1F).

To test whether CXCL12 might itself induce the transcription of CXCL12 in SMCs via an autoregulatory, self-amplifying feedback loop, we treated SMCs with various doses of recombinant CXCL12 (rCXCL12), as well as CM-EPC and EPC-MV containing CXCL12. Indeed, rCXCL12, as well as CM-EPC and EPC-MV, induced an increased expression of CXCL12 mRNA in SMCs. Application of a blocking anti-CXCR4 Ab or CXCR4 receptor antagonist AMD3100 (not shown), but not of an anti-CXCR7 Ab (not shown) significantly inhibited these effects (Figure 1G), demonstrating the role of CXCR4 in self-reinforced CXCL12 expression. 

### 2.2. Higher Concentration of CXCL12 Induce Proliferation of SMCs via CXCR4

**Figure 2:**
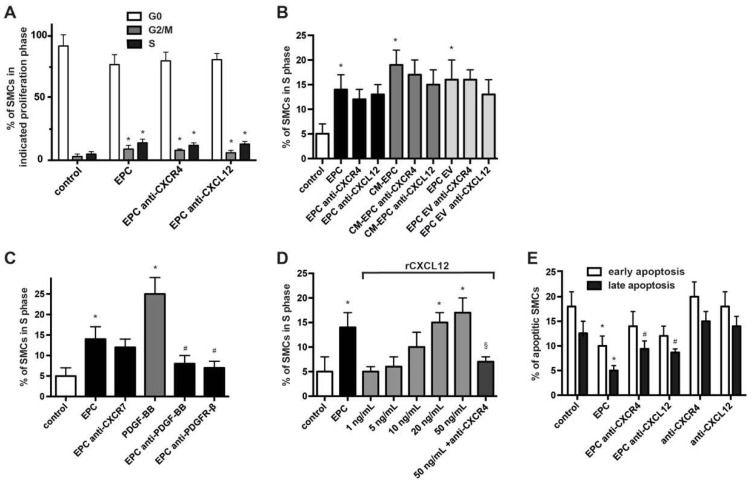
Higher concentration of CXCL12 induce proliferation of SMCs via CXCR4. (**A**) Flow cytometry-based cell cycle analysis of SMCs treated for 24 h with EPCs in the presence or absence of anti-CXCR4 and anti-CXCL12 Abs as indicated. * *p* < 0.05 vs. untreated SMCs (control); *n* = 5. (**B**–**D**) Analysis of SMCs in S phase as determined 24 h after treatment as indicated. * *p* < 0.05 vs. untreated SMCs (control), # *p* < 0.05 vs. EPC treated SMCs in the absence of blocking Abs, § *p* < 0.05 vs. rCXCL12 50 ng/mL treated SMCs; *n* = 4 to 6. (**E**) Annexin V-FITC/PI staining with subsequent flow cytometry analysis to determine the rate of apoptotic SMCs treated as indicated. * *p* < 0.05 vs. untreated SMCs (control) for respective early and late apoptosis, # *p* < 0.05 vs. EPC-treated SMCs for late apoptosis; *n* = 5.

Proliferation and migration of SMCs are crucial processes contributing to atherogenesis and the early stage of neointima formation and EPCs have been shown to modify these capacities of SMCs [6,20]. Proliferation- and cell cycle analysis using BrdU-/7-AAD staining now revealed that inhibition of CXCR4 using blocking Abs or the small-molecule CXCR4 inhibitor AMD3100 (not shown) as well as blockade of CXCL12 using Abs did not relevantly reduce the stimulatory effects of EPCs, CM-EPC and EPC-MV with no significant changes in cell cycle progression and number of cells in S1-phase (Figure 2A,B). Inhibition of CXCR7 as an alternative receptor of CXCL12 also had no modulatory effect, whereas the neutralization of PDGF-BB using blocking Abs or the neutralization of the PDGF-BB receptor PDGFR-β using inhibitory Abs clearly impaired EPC-fostered SMC proliferation (Figure 2C). In control experiments, the presence of blocking CXCR4, CXCL12 or CXCR7 Abs, AMD3100 as well as neutralizing PDGF-BB and PDGFR-β Abs had no effect on the otherwise untreated control SMCs (Supplementary Figure S2). Treatment with rCXCL12 dose-dependently increased the proliferation of SMCs, and inhibition of CXCR4, but not CXCR7, suppressed cell cycle progression and decreased the number of SMCs in S phase (Figure 2D). However, doses of rCXCL12 needed to exceed a threshold value of more than 5 ng/mL to induce such effects.

Combined Annexin V and propidium iodide (PI) staining demonstrated that inhibition of the CXCR4–CXCL12 axis reduced the anti-apoptotic effects of EPCs on quiescent SMCs within 48 h (Figure 2E). However, this effect was only significant for late apoptosis with PI and Annexin V positive SMCs, but not early apoptosis characterized by PI negative and Annexin V positive cells.

### 2.3. EPCs Stimulate Migration of SMCs via CXCL12–CXCR4

**Figure 3:**
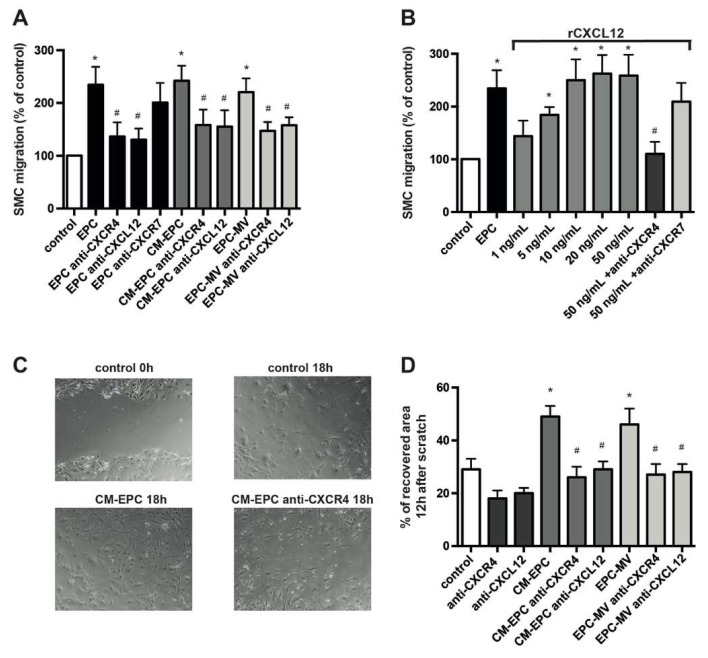
EPCs stimulate migration of SMCs via CXCL12-CXCR4. (**A**,**B**) Transmigration of SMCs as analyzed in transwell chamber experiments with 8 μm pores and expressed as percentage of control. The bottom chamber contained migration medium (DMEM plus 0.5% FBS) supplemented with various doses of rCXCL12, EPCs or their secretory products in the absence or presence of blocking Abs as indicated. * *p* < 0.05 vs. control, # *p* < 0.05 vs. respective treatment in the absence of blocking Abs; *n* = 6. (**C**,**D**) SMC scratch assay. Subconfluent monolayers of SMCs, treated as indicated, were wounded linearly, and the area of the wound subsequently recovered by migrated SMCs was expressed as a percentage of the initial wound area. Representative photomicrographs (**C**) and quantified data (**D**) are shown. * *p* < 0.05 vs. untreated SMCs (control), # *p* < 0.05 vs. respective treatment in the absence of blocking Abs; *n* = 6.

Systematic inhibition of CXCR4 using receptor antagonists and genetic deletion of vascular CXCR4 has been associated with a reduction in SMC content in the neointima of injured murine carotid arteries and in lipid-induced atherosclerotic lesions, whereas the application of EPCs favored increased accumulation of SMCs in the neointima [9,13,14,20,23], suggesting CXCR4 and EPC-dependent alterations in the migratory capacity of SMCs. In transwell chamber experiments, the inhibition of CXCL12 and CXCR4 both reduced transmigration of SMCs seeded in the upper chamber toward EPCs, CM-EPC or EPC-MV placed in the lower chamber (Figure 3A). Blockade of CXCR7 resulted in an impaired but not significantly diminished transfilter migration toward EPCs. Notably, the inhibition of CXCR4, CXCR7 and CXCL12 inhibition showed no effect in regard to migration toward the control medium (Supplementary Figure S3). Presence of rCXCL12 in the lower chamber dose-dependently stimulated the migration of SMCs, and the inhibition of CXCR4 clearly suppressed such effect (Figure 3B). Again, the blockade of CXCR7 resulted in a non-significant reduction of SMC migration toward rCXCL12. In comparison to proliferation, lower doses of rCXCL12 were necessary to alter SMC migration, suggesting a different sensitivity and responsiveness toward CXCL12 for various aspects of SMC behavior.

As a more integrative model for repair after vascular injury, we used an in vitro scratch wound assay with induced linear lesion of the subconfluent SMC monolayer. Interestingly, blockade of CXCR4 or CXCL12 alone impaired wound healing of scratch-injured SMCs in this assay, suggesting a CXCL12–CXCR4-dependent autoregulatory repair mechanism (Figure 3C,D). The addition of CM-EPC or EPC-MV to injured SMCs triggered an accelerated repair of the induced lesion compared to the control. Of note, the magnitude of impairment mediated by inhibition of the CXCL12–CXCR4 pathway was even more pronounced in this scenario.

### 2.4. Proliferation and Migration of Mature Endothelial Cells in Response to EPC/SMC

**Figure 4:**
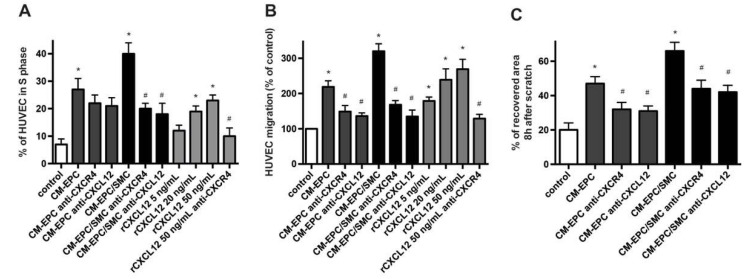
Engagement of CXCL12–CXCR4 in proliferation and migration of endothelial cells. (**A**) Flow-cytometry-based cell cycle analysis of HUVECs treated for 24 h as indicated. * *p* < 0.05 vs. untreated HUVECs (control), # *p* < 0.05 vs. respective treatment in the absence of blocking Abs; *n* = 5. (**B**) Transmigration of HUVECs as analyzed in transwell chamber experiments and expressed as percentage of control. The bottom chamber contained migration medium (DMEM plus 0.5% FBS) supplemented with/without various doses of rCXCL12, CM-EPC or CM-EPC/SMC in the absence or presence of blocking Abs as indicated. * *p* < 0.05 vs. control, # *p* < 0.05 vs. respective treatment in the absence of blocking Abs; *n* = 6. (**C**) HUVEC scratch assay. Monolayers of HUVECs, treated as indicated, were wounded linearly, and the area of the wound subsequently recovered by migrated HUVECs was expressed as a percentage of the initial wound area. * *p* < 0.05 vs. untreated HUVECs (control), # *p* < 0.05 vs. respective treatment in the absence of blocking Abs; *n* = 6.

Reendothelialization is one of the key mechanisms limiting neointima formation after vascular injury, and EPCs are thought to drive endothelial regeneration dominantly by paracrine factors [9,20,24]. We therefore analyzed the effect of the supernatant of EPC–SMC co-cultures (CM-EPC/SMC) on the proliferation and migration of mature endothelial cells. Treatment of HUVECs with CM-EPC/SMC and CM-EPC significantly enhanced their proliferation and migration; however, CM-EPC/SMC triggered a significantly stronger induction of the proliferative and migrative response (Figure 4A,B). In a murine model with arterial injury, selective CXCR4 deficiency on endothelial cells was associated with reduced endothelial cell proliferation and delayed reendothelialization, suggesting a role of the CXCL12–CXCR4 axis in repair processes following vascular injury [9]. Indeed, rCXCL12 also dose-dependently induced both the proliferation and migration of HUVECs (Figure 4A,B). Inhibition of endothelial CXCR4 as well as the blockade of CXCL12 significantly reduced the proliferation and migration rate of HUVECs in response to treatment with rCXCL12 and CM-EPC/SMC. When CM-EPC was added to HUVECs, blockade of the CXCL12–CXCR4 axis conferred a significant inhibitory effect only on the migration but not on the proliferation of endothelial cells. Application of blocking CXCL12 or blocking CXCR4 Abs to otherwise untreated HUVECs had no effect on examined parameters (Supplementary Figure S4). Assessing the wound-healing and repair capacity of endothelial cells in the integrative scratch wound assay, we observed an accelerated repair of the wounded area when CM-EPC or CM-EPC/SMC were added to confluent layers of HUVECs after the induction of injury (Figure 2C). Again, this effect was impaired in the presence of blocking CXCR4 and blocking CXCL12 Abs.

### 2.5. CXCL12–CXCR4-Dependent Control of SMC Phenotype

**Figure 5:**
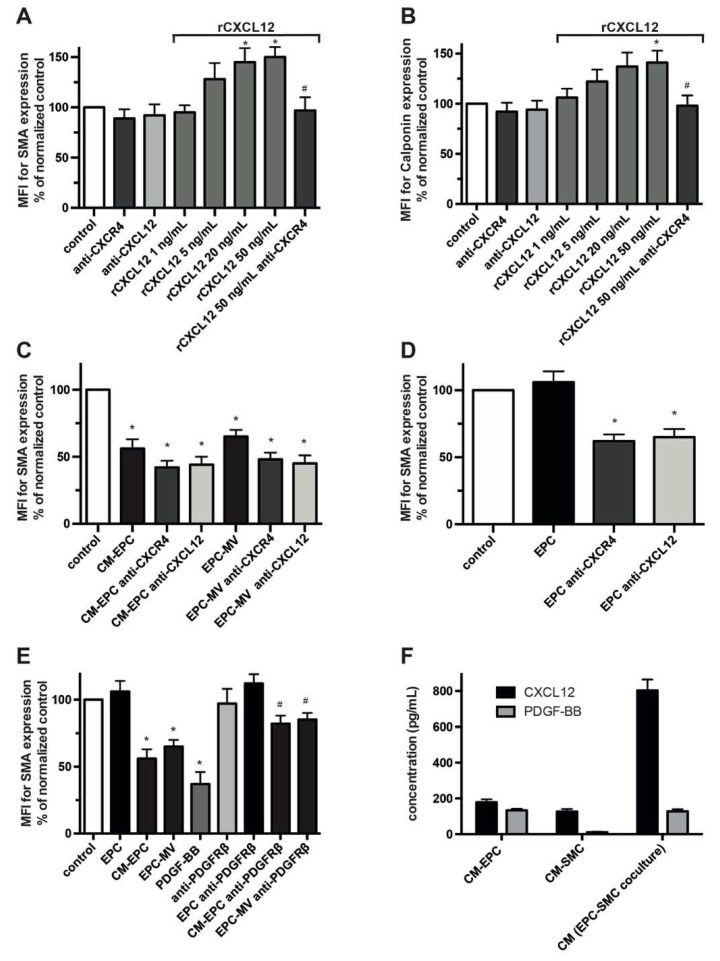
CXCL12–CXCR4-dependent control of SMC phenotype. (**A**–**E**) Analysis of EPC-mediated modulation of SMC phenotype and the involvement of the CXCL12–CXCR4 axis. SMCs were treated as indicated for 48 h and presence of SMA and Calponin was measured using flow cytometry. Data are expressed as mean fluorescence intensity (MFI) in % normalized to untreated SMCs (control). (**A**–**C**) * *p* < 0.05 vs. control and # *p* < 0.05 vs. SMCs treated with rCXCL2 50 ng/mL, (**D**) * *p* < 0.05 vs. SMCs co-cultured with EPCs, (**E**) * *p* < 0.05 vs. control and # *p* < 0.05 vs. the respective treatment of SMCs with CM-EPC or EPC-MV in the absence of anti-PDGFRß; *n* = 4 to 6. (**F**) Comparison of CXCL12 and PDGF-BB released by monocultured EPCs, monocultured SMCs and EPC-SMC co-cultures. Secreted paracrine factors present in the supernatant of the respective cultures were assessed using ELISA; *n* = 5.

Phenotypical changes of SMCs with transformation toward a synthetic phenotype are a hallmark of native and accelerated atherosclerosis crucially contributing to lesion development and neointima formation. In dependence on the mode of interaction, EPCs have been shown to either modulate or preserve the phenotype of SMCs, and vascular lesions of atherosclerotic mice with selective CXCR4 deficiency revealed modified SMC phenotypes [9,14,20]. We now investigated the contribution of the CXCL12–CXCR4 pathway in SMC phenotype control and its involvement in the varying effects of EPCs. As determined by flow cytometry, higher doses of rCXCL12 modulate in a CXCR4-dependent manner the phenotype of SMCs with a tendency toward increased expression of the contractile markers SMA and Calponin (Figure 5A,B). Inhibition of either CXCR4 or CXCL12 had no effect on the phenotype of otherwise untreated SMCs, thus excluding a CXCL12–CXCR4-dependent, self-amplifying feedback loop. Treatment of SMCs with EPC-derived paracrine factors or their MVs for 48 h resulted in a prominent switch toward a more synthetic phenotype with decreased expression of Calponin and SMA (Figure 5C, Supplementary Figure S5A), but the blockade of the CXCL12–CXCR4 axis failed to further modify SMC transformation. In remarkable contrast to secreted EPC factors, the co-cultivation of SMCs with EPCs with enabled direct cell–cell contacts did not confer a reduced expression of markers of a contractile phenotype; however, preceding blockade of CXCR4 on SMCs as well as blockade of CXCL12 resulted in a significant downregulation of the tested markers, collectively reflective for a transition of SMCs toward a more synthetic phenotype (Figure 5D, Supplementary Figure S5B). Inhibition of CXCR7 had no significant modulatory effect in all phenotype experiments (not shown).

The transformative potential of CM-EPC suggests that other EPC-derived paracrine factors are also involved in the regulation of the SMC phenotype and potentially act in opposition to CXCL12 with subsequent downregulation of contractile markers. PDGF-BB, via its receptor PDGFR-β, is a known phenotypic regulator of SMCs and is secreted by EPCs [5,25,26,27]. Inhibition of PDGFR-β using a neutralizing antibody reduced the transformative potential of CM-EPC and EPC-MV but had no effect on SMA expression of SMCs in co-cultivation experiments with EPCs (Figure 5E). In contrast to CXCL12 release, EPC–SMC co-cultivation with established heterocellular contact does not synergistically increase the release of PDGF-BB compared to monocultures [20]. Assessing the CXCL12/PDGF-BB ratio, we found a more than four times higher increase in CXCL12 compared to PDGF-BB in the conditioned medium of EPC–SMC co-cultures when benchmarked to the conditioned medium of EPCs in monoculture (CM-EPC) (Figure 5F). This selective shift in the secretome following EPC–SMC interaction with enabled direct cell–cell contact might contribute to the observed context-dependent effects of EPCs on SMC phenotype.

### 2.6. EPC-Mediated Protection of Cholesterol-Induced Phenotype Switch

**Figure 6:**
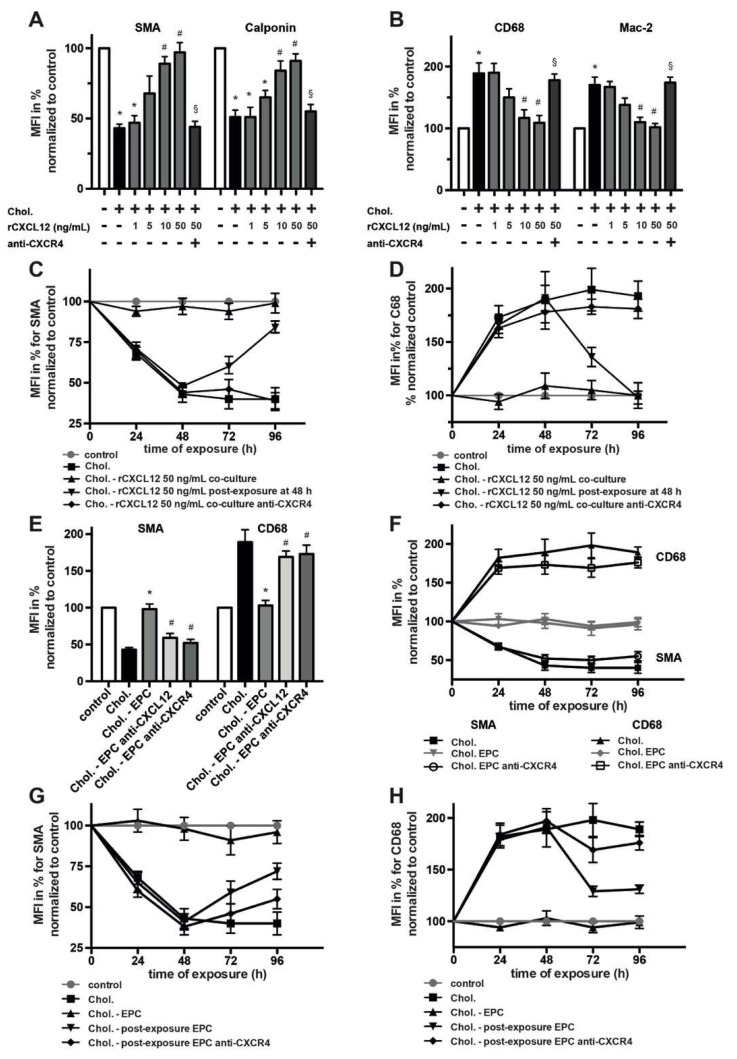
Engagement of CXCL12–CXCR4 in EPC-mediated protection of cholesterol-induced phenotype switch. SMCs were treated as indicated for various time periods and presence of stated phenotype markers was determined using flow cytometry. Data are expressed as MFI in % normalized to untreated SMCs (control). (**A**,**B**) Assessment of the transformative potential of CXCL12 in the presence of SMC loading with Chol:MßCD complexes (Chol). SMCs were treated as indicated for 48 h. Anti-CXCR4 Ab and/or rCXCL12 were added to SMCs simultaneously with Chol:MßCD. * *p* < 0.05 vs. untreated SMCs (control), # *p* < 0.05 vs. SMCs treated with Chol, ^§^ *p* < 0.05 vs. SMCs treated with rCXCL2 50 ng/mL plus Chol:MßCD; *n* = 5. (**C**,**D**,**F**,**G**,**H**) Time course of SMA and CD68 expression by SMCs treated as indicated for various time periods. SMCs loaded with Chol:MßCD were either continuously co-cultured with rCXCL12 or EPCs for up to 96 h or were primary treated with Chol:MßCD for 48 h and only then subsequently exposed to rCXCL12 or EPCs for another 48 h (post-exposure); *n* = 4 to 6. (**E**) Analysis of CXCL12–CXCR4-dependent and EPC-mediated protection of cholesterol-induced phenotype switch after 48 h. * *p* < 0.05 vs. SMCs treated with Chol:MßCD, # *p* < 0.05 vs. SMCs co-cultured with EPCs and treated with Chol:MßCD plus EPCs (Chol.-EPC); *n* = 4.

Exposure of SMCs to cholesterol has been shown to induce transformation with decreased expression of contractile markers and elevated expression of macrophage-related proteins [28,29]. We have previously demonstrated that presence of EPCs in SMC cultures protected from the transformative effect of cholesterol [20], but the underlying mechanism remained to be elucidated. To explore an engagement of CXCL12 and its receptor CXCR4 in such phenotype stabilization, we assessed whether rCXCL12 modulates the transformation of SMCs exposed to cholesterol in two different modalities. In the first set of experiments, rCXCL12 was added simultaneously with Chol:MßCD complexes to subconfluent SMCs, and cells were cultured for various time periods before the analysis of phenotype markers. In this scenario, we observed that, similar to EPCs, rCXCL12 protect SMCs from cholesterol-induced transformation, even at rather low doses (Figure 6A,B). Overall, the presence of rCXCL12 facilitated in a CXCR4-dependent manner retained expression of the contractile markers SMA and Calponin and prevented increased upregulation of the macrophage-related markers CD68 and Mac-2. In the second set of experiments, SMCs were pre-treated with Chol:MßCD complexes for 48 h and only then subsequently exposed to rCXCL12 for another 48 h. In this sequential setting, the addition of rCXCL12 managed to partly reverse cholesterol-induced transformation of SMCs with regained higher SMA expression and the downregulation of CD68 (Figure 6C,D). Notably, both effects were significantly abolished in the presence of blocking CXCR4 Abs (Figure 6A–D) but not blocking CXCR7 Abs (Supplementary Figure S6A,B).

We next evaluated whether the CXCL12–CXCR4 axis is also crucially involved in EPC-mediated protection of SMC phenotype switch upon cholesterol exposure. When SMCs were co-cultured with EPCs in the presence of Chol:MßCD complexes, inhibition of CXCR4 on SMCs as well as blockade of CXCL12 caused a loss of the protective function of EPCs with significant downregulation of SMA and increased expression of CD68 (Figure 6E,F; Supplementary Figure S7A). Again, blockade of CXCR7 had no significant effect in this scenario (not shown). When EPCs were secondarily added to SMCs pre-exposed to Chol:MßCD complexes for 48 h, the expression of the contractile marker SMA by cholesterol-loaded SMCs was partially restored (Figure 6G). Similarly, Chol:MßCD-triggered induction of macrophage-related marker CD68 was partially reverted by post-exposure to EPCs (Figure 6H). These effects were again dependent on CXCR4 (Figure 6G,H) and CXCL12 (Supplementary Figure S7B,C).

## 3. Discussion

The CXCL12–CXCR4 axis is crucially involved in the defense mechanism established to counteract vascular stress and exhibits not only the capacity to recruit and to promote the regenerative potential of EPCs but also the competence to affect lesional SMC accumulation and phenotypic transition [8]. To elaborate the mechanistic link for the recent finding that EPCs may modulate the phenotype of SMCs and increase their neointimal density following vascular injury, and given the well-documented observation that EPCs and SMCs release CXCL12 and express CXCR4 [10,20,25], we analyzed the role of the CXCL12–CXCR4 pathway in the interaction of EPCs and SMCs. Collectively, our data now reveal that CXCL12–CXCR4 signaling is operative in the communication of EPCs and SMCs and hereby triggers an autoregulatory feedback loop for the increased release of CXCL12, mediates the augmentation of the migratory capacity of SMCs as well as protects and favors a contractile over a synthetic phenotype of SMCs. Finally, the interaction of EPCs and SMCs synergistically establishes a microenvironment stimulative for the migration and proliferation of endothelial cells, thus supporting endothelial regeneration.

Given the relevance of CXCL12 for the behavior of EPCs and SMCs as well as processes related to vascular homeostasis [10,30,31], we first evaluated the pattern of CXCL12 expression and release by EPCs and SMCs. We found that, similar to DNA damage and tissue hypoxia [8,32], the mechanical disruption of the SMC network increases CXCL12 release, indicating a stress-triggered induction of CXCL12 secretion. Correspondingly, it has been shown that neointimal SMCs are the main source of CXCL12 in the vessel wall of hypercholesteremic mice after arterial injury [33]. The blockade of CXCL12 or CXCR4 impaired recovery of the disrupted SMC network, suggesting a CXCL12–CXCR4-dependent autoregulatory repair mechanism. Together with the finding of CXCR4-dependent augmentation of EPC recruitment to injured SMCs and the involvement of the CXCL12–CXCR4 axis in EPC-mediated improvement of wound healing and survival of SMCs, these findings endorse the idea of an inherited, CXCL12–CXCR4-based defense and survival program that counteracts processes related to apoptosis and supports the maintenance of vascular integrity. Our experiments with recombinant CXCL12 as well as the co-cultivation studies revealing synergism for the release of CXCL12 demonstrate that EPCs and their secretory products including MVs engage such a reinforcing CXCR4-dependent feedback loop, mediating increased expression and release of CXCL12 by SMCs. According to such concept, the increased and self-amplifying expression of CXCL12 by interacting EPCs and SMCs may act as a homing signal for other CXCR4-positive EPCs, which confer further regenerative signals to the stressed vessel wall, and thus accelerate recovery from injury. In analogy, such self-reinforcing and CXCR4-dependent cycle for CXCL12 expression was observed for mature endothelial cells. Specifically, it was shown that MVs derived from apoptotic endothelial cells may act as a survival factor limiting apoptosis and fostering proliferation of endothelial cells by engaging the CXCL12–CXCR4 pathway via local delivery of miRNA126 and ultimately confer auto-regenerative signals [34]. We now show that a notable amount of CXCL12 generated by EPCs and SMCs is transferred to their respective MVs. Correspondingly, these MVs mediate part of the CXCL12 signaling with subsequent modulation of relevant properties of recipient cells, consistent with the concept of MVs as a delivery system for the intercellular exchange of biological signals and information [21]. Indeed, isolated extracellular vesicles derived from EPCs has been shown to provide tissue protection in various diseases and to beneficially affect vascular regeneration and remodeling, thus paving the way for evaluation of therapeutic application of EPC-MVs [35,36].

In the early stage of vascular lesion development, proliferation and migration of SMCs are the main protagonist driving the expansion and growth of the intima. We confirm previous reports that CXCL12 stimulate cell cycle progression and migration of SMCs via CXCR4 [8,37]. We found that a lower dose of recombinant CXCL12 was necessary to foster migration compared to proliferation, suggesting variable sensitivity and responsiveness of evoked signals and confirming the prominent role of CXCL12 in chemotaxis. Correspondingly, the blockade of the CXCL12–CXCR4 pathway did not significantly alter cell cycle progression induced by EPCs but considerably reduced EPC-triggered migration and wound healing of scratch-injured SMCs. In addition to the possibility that levels of released CXCL12 during the interaction of SMCs with EPCs or their secretory products are below the threshold limit value to substantially induce SMC growth alone, this finding may also indicate that redundant EPC-derived signals are operative and sufficient in the regulation of increased SMC proliferation. Indeed, EPCs harbor an abundance of paracrine factors with the potential to orchestrate growth of neighboring SMCs [25,38,39]. Of note, conclusions drawn by direct comparisons of applied or present CXCL12 concentration in experiments with recombinant CXCL12 and EPCs or their secretory factors should be drawn with caution, as contribution of CXCL12 and its receptor CXCR4 may crucially depend on the distinct molecular milieu at the site of action, which relevantly influence responsiveness and sensitivity toward CXCL12–CXCR4 signaling due to ligand diversity, the regulation of receptor availability, receptor isoform and oligomerization [10,30,40]. Collectively, heterogenous constellations may differentially regulate the signaling pathways, resulting in variable threshold concentrations triggering examined effects.

Maintenance of the endothelial integrity and reendothelialization are of paramount importance for limiting atherosclerotic lesion progression and neointima formation after vascular injury [24,25]. In mouse models with arterial injury the degree of reendothelialization showed an inverse correlation with neointima formation, suggesting that expedited recovery of the endothelial lining might establish a crucial stop signal for further expansion of the neointima [9]. Numerous studies demonstrated that EPCs contribute to vascular repair, address endothelial dysfunction and support the restoration of the endothelium [17,41,42]. Mechanistically, it is believed that EPCs convey such effects dominantly via paracrine factors and MVs, inducing the promotion of regenerative processes operated by mature endothelial cells and resident vascular progenitor cells [24]. The maintenance of the endothelial barrier function has been shown to be dependent on CXCL12-mediated vascular CXCR4 signaling, which might engage WNT/ß-catenin pathways to promote VE-cadherin expression and stabilization of junctions [9,14]. Conversely, disruption of this signaling pathway by genetic deletion of vascular CXCR4 increases endothelial permeability and is permissive for the augmented recruitment of inflammatory cells, potentially driving the progression of atherosclerosis. In light of these findings, we now show that conditioned medium from EPC-SMC co-cultures, containing a synergistically increased CXCL12 concentration, is superior to foster CXCR4-dependent proliferation, migration and wound healing of endothelial cells when compared to conditioned medium derived from EPCs in monoculture. CXCL12–CXCR4 signaling may therefore establish the foundation for a mutually reinforcing alliance of recruited EPCs interacting with SMCs to confer accelerated restoration of the endothelial barrier.

Lineage-tracing studies and single-cell transcriptomics confirmed that SMCs take center stage in all steps of post-injury remodeling and atherosclerosis, revealed the diversity of SMC-derived lesion cells, and refined the importance of clonality and phenotype switch in lesion development [4,6]. Given that SMCs constitute the major cell type in the lesion and dominantly provide extracellular matrix components [6], their phenotypic modulation might crucially determine the inflammatory state of the blood vessel, evoke changes in the molecular milieu to adjust the behavior of other recruited and resident cells and together with oligoclonal expansion influence the progression and stability of the nascent lesion [6,29]. Phenotype switching of contractile SMCs to a synthetic phenotype, in particular with macrophage-like features, has long been considered to actively contribute to atherogenesis and neointima formation. We now show that heterocellular signals relevant for conveying preservation of a contractile SMC phenotype critically involve the engagement of CXCL12 and its receptor CXCR4 during direct EPC–SMC interaction with established cell–cell contact. In line with this finding, atherosclerotic lesions in hypercholesteremic mice with SMC-specific CXCR4 deficiency displayed an amplified switch of SMCs to a synthetic, macrophage-like and less contractile phenotype [14]. Considering that the balance of transformative effects is contrarily shifted when direct contact of EPCs and SMCs is not established [20], our data suggest that some factors present in the CM-EPC and EPC-MV counteract this protective, CXCL12–CXCR4-mediated mechanism and propagate the downregulation of contractile markers. EPCs are known to release an orchestra of potent agents that are candidates to modulate the phenotype of SMCs, including PDGF-BB as a promoter of the synthetic phenotype [26,38]. Since the secretome of EPCs underlies dynamic modifications reflecting alterations of environmental cues [25,43], we now exemplarily documented a divergent adjustment for the release of CXCL12 and PDGF-BB caused by co-cultivation with SMCs. The selective shift in the CXCL12/PDGF-BB ratio following EPC–SMC interaction might contribute to the observed context-dependent effects of EPCs on SMC phenotype. Additionally, the lack of feedback communication between cells and missed opportunity for juxtracrine signaling in experiments with isolated paracrine factors might constitute a possible explanation for the differential effects.

Accumulation of foam cells constitutes a hallmark of native atherosclerosis and feature of advanced stages of accelerated atherosclerosis [6,29]. Genetic lineage-tracing studies provided evidence that a substantial proportion of lesional foam cells are derived from phenotypic transformed SMCs expressing macrophage markers such CD68 and Mac-2 [4,6]. Exposure to cholesterol has been linked to the transdifferentiation of SMCs to a macrophage-like state [20,28]. Time-course experiments now revealed that CXCL12 not only stabilizes the contractile phenotype of SMCs via CXCR4 but also reverses the phenotypic switch upon exposure to cholesterol. Most notably, we demonstrate that EPCs effectively engages this CXCL12–CXCR4 pathway to confer their protective function on the phenotype of SMCs with retained expression of SMA and prevented the upregulation of CD68 despite the presence of cholesterol. Via CXCL12, EPCs may also restore the contractile phenotype of once transdifferentiated and cholesterol-exposed SMCs. These results reveal that EPCs are relevantly invested in phenotypic homeostasis of SMCs via CXCL12–CXCR4 signaling. By averting a phenotypic switch of SMCs, even under adverse conditions such as presence of cholesterol, EPCs attenuate processes leading to the adoption of a macrophage-like state, where SMCs would produce inflammatory mitogens and cytokines, increasingly attract immune competent cells and drive pathological progression of the lesion. By engaging vascular CXCR4 signaling, EPCs may therefore confer a protective function to hamper foam cell formation and retain the stability of the lesion.

In the context of injury-driven atherosclerosis, we have previously shown that effects of EPCs on SMCs are ambivalent; however, EPC-mediated phenotype control and the acceleration of reendothelialization appear to dominate over the stimulating effects on SMC proliferation and migration, collectively resulting in a reduction in neointima formation following application of EPCs [20]. Our data now suggest that engagement of CXCL12–CXCR4 signaling during EPC–SMC interaction critically modulates SMC behavior and may thus confer alteration in lipid-induced atherosclerosis and postinjury vascular remodeling. According to this concept, vascular stress with injury of SMCs unleashes a CXCL12–CXCR4-based autoregulatory feedback loop constituted by the local accumulation of CXCL12 via self-amplifying release by SMCs and via the activation of a reinforcing, synergistic CXCL12 secretion during EPC–SMC interaction, augmented homing of CXCR4-expressing EPCs programmed to accelerate vascular recovery, attenuated apoptosis and enhanced wound healing of SMCs and, finally, EPC-mediated and CXCL12–CXCR4-promoted protection and stabilization of SMC phenotype. These processes may support the maintenance of vascular integrity, prevent the exacerbation of proinflammatory pathways and therefore beneficially modify the composition and stability of the vessel wall. Considering the ambivalent contributions of the CXCL12–CXCR4 axis to regenerative vascular processes and the complex regulation of the CXCL12–CXCR4 pathway, further studies are necessary to decipher the functional consequences in various disease settings and to establish beneficial therapeutic interventions [30,44].

## 4. Materials and Methods

### 4.1. Cell Isolation and Culture

Isolation and culture of EPCs and human umbilical vein endothelial cells (HUVECs; PromoCell, Heidelberg, Germany) were performed according to established protocols [25,41]. Briefly, peripheral blood mononuclear cells (PBMCs) were separated by Biocoll density gradient centrifugation (Biochrom, Berlin, Germany) from buffy coats of healthy human donors and plated on fibronectin-coated 24-well plates (5 × 10^5^/well) in MV2 endothelial growth medium (PromoCell). Medium was changed at day 4, and cells were used for experiments at day 7. PBMCs cultured under these specific conditions developed a spindle-shaped appearance, formed typical cell clusters, intensely took up acetylated LDL and bound endothelial specific lectin (not shown). These cells with features of endothelial and myeloid cells were identified as endothelial progenitor cells (EPCs). Human aortic vascular smooth muscle cells (SMCs; Lonza, Basel, Switzerland) cultured using Smooth Muscle Cell Growth Medium 2 (PromoCell) were used at passage 4 to 8, however, SMCs for phenotype analysis were restricted to passage 4. HUVECs were used at passage 3 to 5 and cultured in endothelial cell growth medium (PromoCell). For preparation of conditioned medium (CM), EPCs and SMCs cultured in 24-well plates at 5 × 10^5^ cells/well were washed twice with PBS to remove residual growth factors and cultured in 500 μL DMEM medium plus 0.5% FBS. After 24 h, the medium was collected and subsequently centrifuged at 1250 g for 10 min to remove cell debris. DMEM medium plus 0.5 % FBS served as a control (unconditioned medium). Extracellular vesicles derived from EPCs and SMCs were isolated from CM-EPC and CM-SMC by gently passing the conditioned medium through a 0.8 μm filter and subsequent centrifugation at 20,000× *g* for 20 min. As assessed by flow cytometry, such protocol resulted in the isolation of larger-sized vesicles commonly referred as microvesicles (MVs) or microparticles. Scanning electron microscopy (ScEM; FEI/Philips ESEM XL30 FEG, Amsterdam, Netherlands), performed as previously described [25], confirmed MV secretion and revealed that the majority of generated MVs measured 200 to 800 nm (Supplementary Figure S1). The pellet obtained by centrifugation was resuspended in 0.22 μm filtered DMEM medium plus 0.5% FBS with a volume equivalent to the volume of CM-EPC and CM-SMC. When needed, 1.5 mM calcium was added for APC-labeled Annexin V staining (BD Pharmingen, Franklin Lakes, NJ, USA). Flow cytometry was used for characterization of MVs. Application of size standard beads with a diameter of 0.5 and 1.0 μm (Nanobead and Microbead NIST Traceable Particle Size Standards, Polysciences, Warrington, PA, USA) allowed estimation of size distribution and gating from the resolution limit of the flow cytometer to 1.0 μm. For enumeration, MV samples were added to tubes with a known number of 4.2 μm calibrated microbeads (Trucount beads, BD Biosciences, Franklin Lakes, NJ, USA), thus allowing for quantitative determination of MVs. EPC-MV and SMC-MV were determined as a count of Annexin V positive particles with a size ≤ 1.0 μm. To address concerns regarding swarm detection of smaller sized microvesicles, adequate dilution of the samples and a low flow rate was chosen. According to this quantification method, we measured 12,900 ± 3200 EPC-MV/μL and 4100 ± 1400 SMC-MV/μL and purity of MVs was >99%. Calibrated to the amount of donor cells, the obtained MV concentration correlates on average to 12.9 EPC-MV per donor EPC and to 4.1 SMC-MV per donor SMC. Analysis of surface markers with labeled antibodies (Biolegend, San Diego, CA, USA) identified high expression of CD31, CD45 and CXCR4 (each > 95%) and relevant expression of KDR (60%) by EPC-MV, confirming that they carry membrane receptors that are also characteristic for PBMC-derived cultured EPCs.

### 4.2. ELISA and RT-PCR for CXCL12

Concentrations of CXCL12 and PDGF-BB were determined with the Duo-Set ELISA Development Kit (R&D Systems, Minneapolis, MN, USA). Thoroughly washed SMCs and EPCs suspended in DMEM plus 0.5% FBS were seeded in 96-well plates either as monoculture or as EPC–SMC co-culture, and supernatant was analyzed after 24 h. The number of seeded cells per well and the applied volume for the 96-well plates were matched to the 24-well plates used for generation of CM and MVs. For detection of CXCL12 in MVs, vesicles were isolated as described from the supernatant and subsequently lysed in RIPA buffer before analysis. Real-time RT-PCR was performed with the QuantiTect SYBR-Green PCR kit (Opticon MJ Research, Hercules, CA, USA) or Taqman Gene Expression Master Mix (Applied Biosystems, Waltham, MA, USA) and ready-to-use CXCL12 primers (Assay ID: Hs00171022_m1 for CXCL12; Applied BioSystems). Gene expression was normalized to GAPDH using the ΔΔCt method. All assays were performed in triplicate.

### 4.3. Cell Proliferation Assay

For assessment of proliferation and cell cycle analysis, we used a flow cytometric approach to determine the incorporation of 5-bromo-2′-deoxyuridine (BrdU) and staining for 7-AAD according to the manufacturer’s protocol (FITC BrdU Flow Kit, BD Biosciences). In brief, SMCs or HUVECs rendered quiescent by serum starvation for 24 h were seeded in 96-well plates (~50% confluent cells) and subsequently treated with EPCs (5 × 10^4^), 50 μL CM-EPC, 50 μL CM-EPC/SMC, EPC-MV, recombinant human PDGF-BB (20 ng/mL, R&D Systems) or recombinant human CXCL12 (rCXCL12; 1 to 50 ng/mL, R&D Systems) as indicated. In some experiments, blocking antibodies (Abs) targeting CXCL12 (MAB310; R&D Systems), CXCR4 (MAB171; R&D Systems), CXCR7 (MAB42273; R&D Systems), PDGF-BB (AB-220; R&D Systems), the small molecule CXCR4 receptor antagonist AMD3100 (Sigma-Aldrich) or respective isotype controls were applied. BrdU, which is incorporated into newly synthesized DNA strands of actively proliferating cells, was added 1 h after cell seeding at a final concentration of 10 μM. For differentiation of cells, samples were stained with APC-labeled CD31 (R&D Systems). SMCs were distinguished from co-cultivated cells by their FSC/SSC profile and absent staining for CD31. Cell-cycle progression was detected on a FACSCanto II flow cytometer (BD Biosciences) and was analyzed by FlowJo software (Tree Star Inc., Ashland, OR, USA).

### 4.4. Apoptosis Assay

Apoptosis of SMCs, left untreated or treated with EPCs, was measured using the Apoptosis Detection kit I (BD Biosciences) per manufacturer’s recommendations and performing flow cytometry. As a standard, 1 × 10^5^/mL of cells per treatment condition were fixed and stained with 5 μL Annexin V–FITC and 5 μL propidium iodide (PI). Using flow cytometry, EPCs and SMCs were discerned and separately gated based on FSC/SSC profile and expression of the EPC surface marker CD31. Early apoptotic cells were defined as PI negative and FITC Annexin V positive cells, late apoptotic cells were defined as PI and FITC Annexin V positive cells.

### 4.5. Transwell Migration Assay

SMC and HUVEC migration were evaluated in 24-well modified Boyden chambers (Sigma-Aldrich, St. Louis, MO, USA) with 8 μm pore-size filters. EPCs, CM-EPC, CM-EPC/SMC, EPC-MV or various concentrations of rCXCL12 (1 to 50 ng/mL) were suspended in migration medium (DMEM plus 0.5% FBS) and placed in the lower chambers. In some experiments, blocking Abs targeting CXCR4, CXCR7 or CXCL12 were added. SMCs or HUVECs starved for 24 h were seeded in the upper chamber at densities of 5 × 10^4^ cells per well in migration medium and were allowed to migrate in the incubator for 6 h. Finally, the membrane was transferred onto a coverslide, and cells were stained using Vectashield mounting medium containing 4,6-diamidino-2-phenylindole (DAPI; Vector Laboratories, Burlingame, CA, USA). Four randomly chosen fields per well were photographed and the number of migrated cells on the side which faced the EPCs/chemoattractive agent was manually counted.

### 4.6. Scratch Wound Assay

A scratch assay was used to analyze the migratory and proliferative capacity of SMCs and HUVECs in an in vitro injury model. Cells were grown to subconfluence in 24-well plates and the cell monolayer was wounded linearly with a pipette tip (2 parallel scratches per well) generating a gap of approximately 1 mm width. Cells were gently washed two times and continuously cultured in the unconditioned/conditioned medium or with EPC-MV. Again, in some experiments, blocking Abs were applied to test the involvement of CXCL12, CXCR4 and CXCR7. To evaluate the recovery of the scraped wounds, microscopic photographs were taken at indicated intervals, and the recovered area was quantified.

### 4.7. Flow Adhesion Assay

Laminar flow adhesion assays were performed as described. Ten dishes were coated with confluent layers of SMCs and assembled as the lower wall of a flow chamber on stage of an Olympus IX50 microscope. When noted, SMCs were stimulated with low-dose TNFα (1 ng/mL, Peprotech, London, UK) and/or scratch injured 24 h before perfusion of EPCs. EPCs were resuspended in the presence or absence of a blocking CXCR4 Ab for 30 min in HHMC-medium (1.5 × 10^5^ cells/mL) and perfused with 1.5 dyne/cm^2^ at 37 °C. After 4 min, firmly adherent cells were quantified in multiple fields recorded with a JVC 3CCD video camera.

### 4.8. SMC Phenotype Analysis

SMC phenotype characterization was performed by flow cytometry. Subconfluent SMCs at passage 4 were left untreated or treated with EPCs, CM-EPC, EPC-MV, PDGF-BB (20 ng/mL; R&D Systems) or various concentrations of rCXCL12 for up to 96 h before fixation and analysis. When indicated, cells were cultured in the presence of blocking Abs targeting CXCR4, CXCL12, CXCR7 or PDGFRß (AF385; R&D Systems). For cholesterol loading experiments, SMCs were treated with 10 μg/mL cholesterol:methyl-ß-cyclodextrin complexes (Chol:MßCD; Sigma-Aldrich) with 0.2% BSA for up to 96 h. For preparation of staining, SMCs were incubated for 20 min at 4 °C in BD Cytofix/Cytoperm (BD Biosciences) to fix the cells and permeabilize the cell membrane. Cells were then washed with 10% BD Perm/Wash Solution and incubated with antibodies against α-Smooth Muscle Actin (SMA; R&D Systems), Calponin or CD68 (all Abcam, Cambridge, UK) for 30 min. Following washing in 10% BD Perm/Wash Solution, primary Abs were detected using FITC-labeled or APC-labeled anti-mouse or anti-rabbit secondary Abs (Sigma-Aldrich). Mac-2 on the surface of SMCs was detected by an APC-conjugated antibody (R&D Systems). Isotype-matched IgG1 or IgG2a used as a negative control were purchased from the same manufacturer as the immune Abs.

### 4.9. Statistical Analysis

Data represent mean ± SD and were either analyzed by Student’s *t*-test and one-way ANOVA followed by Newman–Keuls post-hoc test (for normally distributed data) or by nonparametric Mann–Whitney test and Kruskal–Wallis test with post-hoc Dunn test (for non-normally distributed data) using the GraphPad Prism version 8 (GraphPad Software, San Diego, CA, USA) as appropriate. Differences with *p* < 0.05 were considered to be statistically significant. The number of independent experiments is stated in the figure legends.

## Data Availability

The data presented in this study are available on request from the corresponding author.

## Supplementary Materials

The following supporting information can be downloaded at: https://www.mdpi.com/article/10.3390/ijms23020867/s1.
