# Supplementary material for: Engagement of the CXCL12–CXCR4 Axis in the Interaction of Endothelial Progenitor Cell and Smooth Muscle Cell to Promote Phenotype Control and Guard Vascular Homeostasis
Source: Int J Mol Sci. 2022 Jan 14;23(2):867. doi: 10.3390/ijms23020867 (PMC8776104; doi:10.3390/ijms23020867)
Supplement: Supplementary file 1 [file ijms-23-00867-s001.zip › ijms-1504888-supplementary.pdf]

International Journal of *Molecular Sciences*

Supplementary Figures:

Supplementary Figure S1

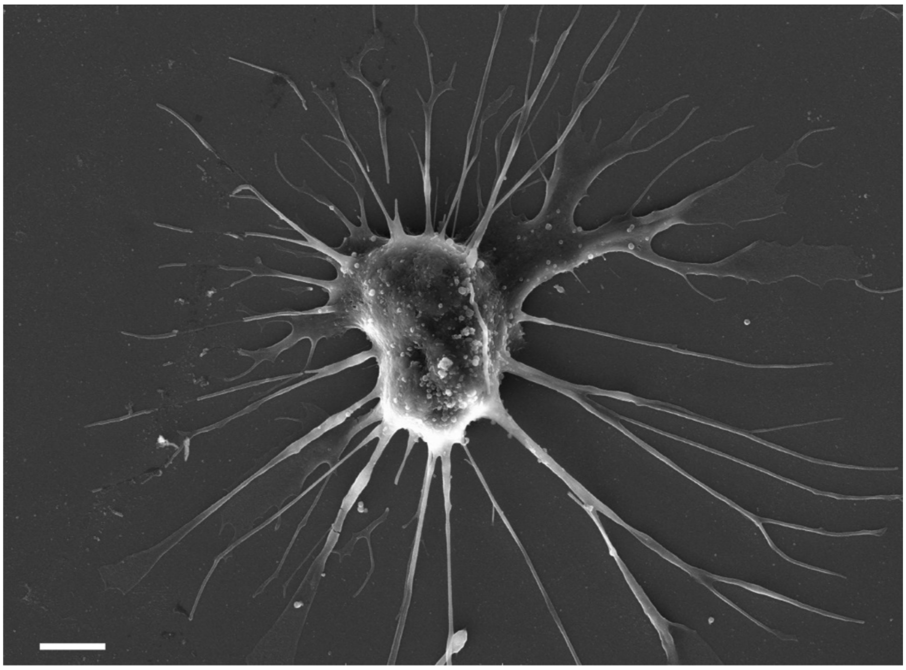

Supplementary Figure S1. Scanning electron microscopy (ScEM) for detection of EPC-derived microvesicles. EPCs were plated on glass coverslides and fixed with 3% glutaraldehyde (GA). Following post-fixation with 1% GA, dehydrated samples were processed by critical point drying with CO^2 and sputter-coated with gold. Shown is a representative ScEM image of a EPC grown on glass coverslides. Scale bar= 1000 nm

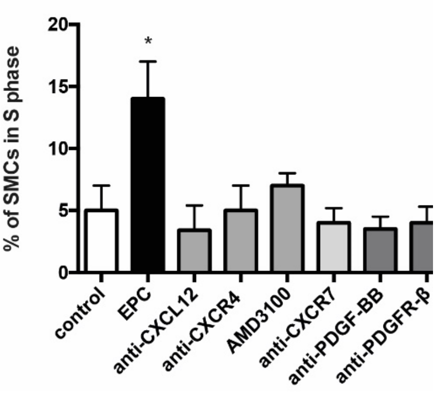

Supplementary Figure S2. EPCs induce enhanced SMC proliferation. Flow cytometry-based cell cycle analysis of SMCs treated for 24 h with EPCs, blocking Abs or AMD3100 as indicated. * P<0.05 vs untreated SMCs (control); n=5.

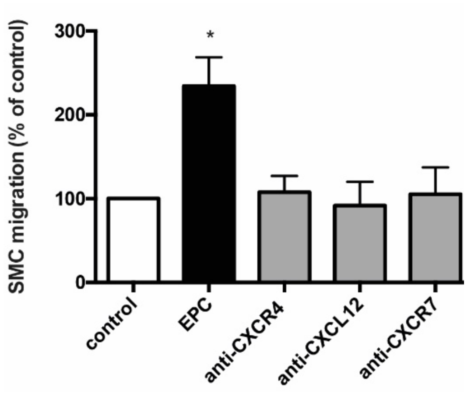

Supplementary Figure S3. EPCs stimulate migration of SMCs. Transmigration of SMCs as analyzed in transwell chamber experiments with 8 µm pores and expressed as percentage of control. The bottom chamber contained migration medium (DMEM plus 0.5% FBS) supplemented with EPCs or blocking Abs as indicated. * P<0.05 vs untreated SMCs (control); n=6.

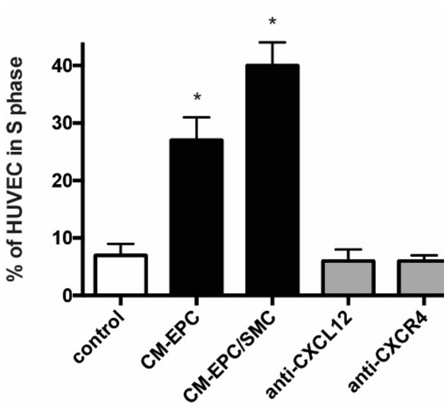

Supplementary Figure S4. Proliferation of endothelial cells promoted by EPC/SMC. Flow cytometry-based cell cycle analysis of HUVECs treated for 24 h as indicated. * P<0.05 vs untreated HUVECs (control); n=5.

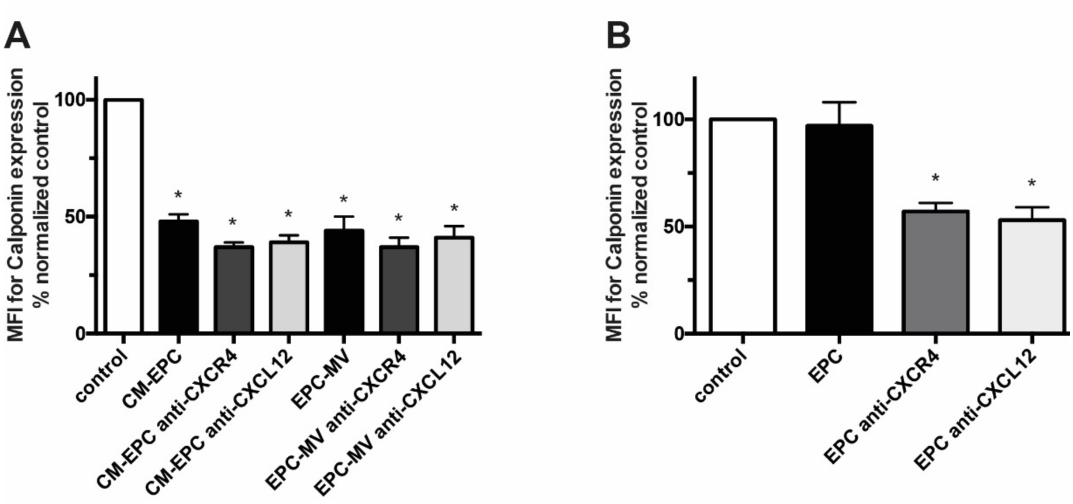

Supplementary Figure S5. Analysis of SMC phenotype. SMCs were treated as indicated for 48 h and presence of Calponin was measured using flow cytometry. Data are expressed as mean fluorescence intensity (MFI) in % normalized to untreated SMCs (control). (**A**) * P<0.05 vs untreated SMCs (control), (**B**) *P<0.05 vs SMCs co-cultured with EPCs; n=5.

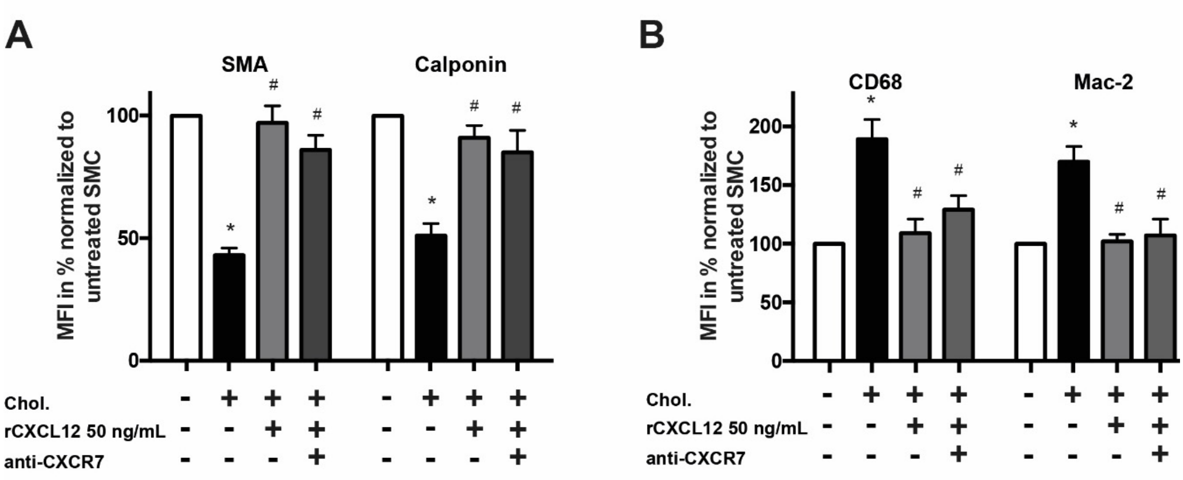

Supplementary Figure S6. Role of CXCL12 in protection of cholesterol-induced SMC phenotype switch. SMCs loaded with or without Chol:MßCD complexes (Chol) were treated as indicated for 48 hours and presence of stated phenotype markers was determined using flow cytometry. Data are expressed as MFI in % normalized to untreated SMCs (control). (**A**) * P<0.05 vs untreated SMCs (control), (**B**) # P<0.05 vs SMCs treated with Chol; n=5.

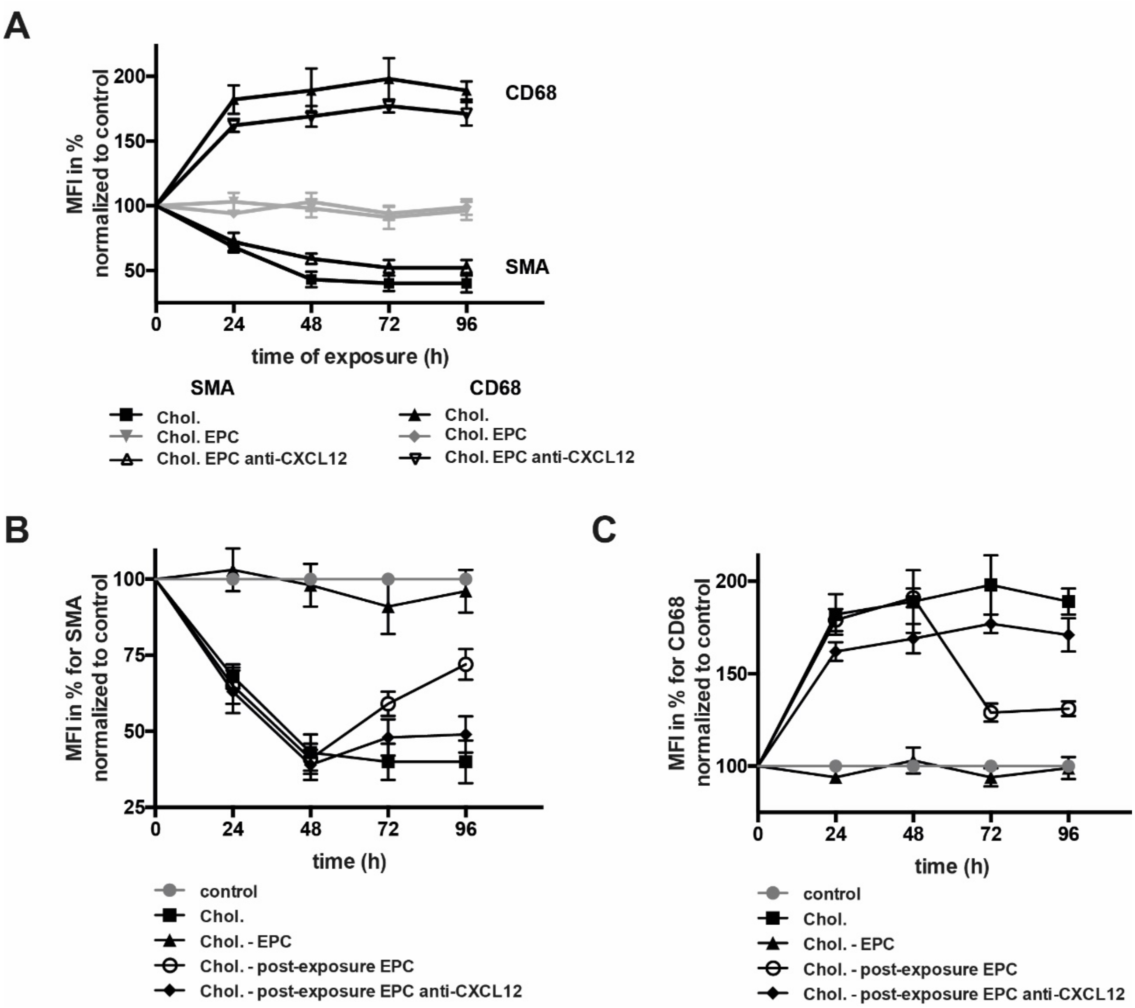

Supplementary Figure S7. Engagement of CXCL12 in EPC-mediated reversal of cholesterol-induced SMC phenotype switch. (**A** to **C**) Time course analysis of SMA and CD68 expression by SMCs treated as indicated for various time periods. (**B**, **C**) SMCs loaded with Chol:MßCD were either continuously co-cultured with EPCs for up to 96 h or were primary treated with Chol:MßCD for 48 h and only then subsequently exposed to EPCs in the presence of absence of a blocking CXCL12 Ab for another 48 h (post-exposure); n=4.
